# Genotypic Diversity, Antibiotic Resistance and Bacteriocin Production of Enterococci Isolated from Rhizospheres

**DOI:** 10.1264/jsme2.ME12041

**Published:** 2012-10-31

**Authors:** Naouel Klibi, Naouel Ben Slimen, Imen Fhoula, Maria López, Karim Ben Slama, Daniele Daffonchio, Abdellatif Boudabous, Carmen Torres, Hadda Ouzari

**Affiliations:** 1 Laboratoire de Microorganismes et Biomolécules actives, Département de Biologie, Faculté de Sciences de Tunis, Campus Universitaire, 2092 Tunis, Tunisia; 2 Area de Bioquímica y Biología Molecular, Universidad de La Rioja, 26006 Logroño, Spain; 3 Dipartimento di Scienze e Tecnologie Alimentari e Microbiologiche, Università degli Studi, 20133, Milano, Italy

**Keywords:** Enterococci, antibiotic resistance, bacteriocins, MLST, PFGE

## Abstract

This study aimed to identify and to characterize rhizospheric-derived enterococci. The results showed the prevalence of *Enterococcus faecium* species (97%) vs. *Enterococcus durans* (3%). Susceptibility testing for antibiotics showed a low percentage of resistance to erythromycin (3.2%) and tetracycline (11.2%), and intermediate resistance to vancomycin (6.5%). Nevertheless, a high proportion of bacteriocin production was recorded. Furthermore, PCR detection of antibiotic resistance and bacteriocin production-encoding genes was investigated. Pulsed-field gel electrophoresis typing (PFGE) showed a great variability of enterococci in the rhizosphere. Moreover, mutilocus-sequence-typing analysis (MLST) revealed the identification of three new sequence types (STs), which were registered as ST613, ST614 and ST615.

Soil is a vast reservoir for microorganisms. Many bacteria have been found in soils, especially in the rhizosphere, and the abundance in vegetated soils is due to the availability of nutrients via plant roots and to the diversity of ecological niches (16).

*Enterococcus* spp. colonizes the gastrointestinal tract of many animals and is also commonly found in soil, plants, vegetables and water (1, 2, 31, 32). Together with coliform bacteria, enterococci are considered as an indicator of fecal contamination (21); however, other studies have suggested that these bacteria were likely derived from environmental sources unrelated to fecal contamination. According to Mundt *et al.* (33, 34), enterococi also occur on plants in a truly epiphytic relationship and are able to spread by seeds and reproduce on growing plants. *E. faecium* is the enterococcal species with the highest level of resistance to antibiotics compared to other species (26). The high use of antibiotics in human and veterinary medicine, livestock, aquaculture, and agriculture, associated with the diverse mechanisms of bacterial genetic transfer, could cause antibiotic molecules, antibiotic-resistant bacteria and/or their resistance genes in different habitats (14, 23, 26, 36, 45). Enterococci are intrinsically resistant to semi-synthetic penicillins, aminoglycosides (low level), vancomycin (low level for some species such as *E. gallinarum*, *E. casseliflavus* and *E. flavescens*), lincosamides (most), polymyxines, streptogramin A (*E. faecalis*) and monobactams (13, 35). In addition, they are able to acquire multi-resistance to many antibiotics (1, 37, 39, 42, 43).

The problem of antibiotic resistance in enterococci is not only restricted to the clinical setting but also to other environments, such as the intestinal tract of healthy humans, sewage water and food-producing animals (4, 38, 41). Little is known about antibiotic resistance in enterococci in the natural environment, especially in soil. The main objectives of the current study were to determine the taxonomy and genetic diversity of enterococci in the rhizosphere ecosystem and to evaluate their content in antibiotic resistance and virulence genes, and also to evaluate their antibiotic resistance pattern and their ability to produce bacteriocins.

Enterococcal isolates were recovered from 31 rhizospheric samples of olive plants obtained from eight different regions in the south and north of Tunisia. Although the major part of the country is characterized by a sub-arid climate, the northern regions differ mainly by darker soil and more abundant rainfall than in the southern parts. Enterococci were isolated by the enrichment method according to the procedure of Zamudio-Maya *et al.* (47), with some modifications. Briefly, 1 g sample was resuspended in 5 mL MRS broth and incubated at 30°C for 2–3 days. The obtained cultures were diluted and spread onto MRS agar medium supplemented with 0.01% (w/v) bromocresol green to enhance the morphological differentiation of lactic acid bacterial colonies. Entrococcal isolates were identified to the genus level by biochemical methods (Gram staining, bile-esculine and hypersaline reaction) and to the species level by molecular PCR assays using specific primers for *ddl_E. faecalis_*, *ddl_E. faecium_*, *vanC-1*, *vanC-2*, *mur-2eh* and *mur-2ed* genes specific for *E. faecalis*, *E. faecium*, *E. gallinarum*, *E. casseliffavus*, *E. hirae* and *E. durans*, respectively, as previously described (5, 12, 30).

Antibiotic susceptibility testing was performed by the disk diffusion method on BHIA following previously reported criteria (CLSI 2010). The antibiotics tested (Bio-Rad Laboratories, Hercules, CA, USA) were as follows: erythromycin, ampicillin, vancomycin, teicoplanin, streptomycin, gentamicin, tetracycline, chloramphenicol, trimethoprim-sulfamethoxazol and pristinamycin. High-level aminoglycoside resistance was detected using high-charge disks of gentamicin (Gen, 120 μg) and streptomycin (Strep, 300 μg). MICs for vancomycin and streptomycin were also determined using broth dilution and agar dilution methods (CLSI 2010). *E. faecalis* ATCC 29212 was used as the quality control strain for all susceptibility testing.

Genomic DNA of enterococci was obtained using a commercial solution for DNA extraction “Instagene matrix” (BioRad). The genes studied were as follows: *erm*(A), *erm*(B), *erm*(C), *erm*(T), *msr*(A), *tet*(M) and *tet*(L) encoding resistance to erythromycin or tetracycline (43). Vancomycin resistance mechanisms were analyzed by PCR using specific primers for amplification of *van*A, *van*B, *van*C-1, *van*C-2/3 and *van*D genes (28), and Taq polymerase (Bioline, Labolan, Spain). For vancomycin-resistant strains negative for the above referred genes, new PCR were performed with degenerated primers in order to amplify different D-Ala ligases: *van*V1 (GGIGAAGATGGITCITTICAAGG) and *van*V2 (IGTAAAICCIGGIATIGTATT), (12); *van*V3 (GAR GATGGITSCATMCARGGW) and *van*V4 (MGTRAAICCIGGCAKRGTRTT), (7) and N*van*F (GTTTGGGGGTTGCTCAGAGG) with N*van*R (TCACCCCTTTAACGCTAATACGATC), and the obtained amplicons were sequenced (46).

The presence of *hyl* and *esp* genes, encoding virulence factors (glycosil-hydrolase and enterococcal surface protein, respectively), were studied by PCR (40). All PCR reactions included positive and negative controls from the University of Rioja (Spain).

The antimicrobial activity of bacterial cultures was assayed using the toothpick method previously described (11). Briefly, 50 μl of an overnight culture in BHI broth of the indicator strains (*Listeria monocytogenes* C137, *E. gallinarum* C86, *E. faecium* AR1, *E. faecium* AR36, *E. faecium* AR58, *E. faecalis* AR42 and *E. faecalis* AR69) was added to 5 mL molten Tryptic Soy Broth (Becton Dickinson, Franklin Lakes, NJ, USA), supplemented with 0.7% (w/v) agar and 0.5% (w/v) yeast extract, mixed and poured onto a yeast extract-supplemented Tryptic Soy Agar (Becton Dickinson) plate. A single colony of each isolate was transferred with a sterile toothpick to the agar plate previously seeded with the indicator microorganism. The plates were then incubated at 37°C for 24 h. Antimicrobial activity was visually detected by the presence of clear inhibition zones around the producer strains (only inhibition halos with diameters higher than 3 mm were considered as a positive result). Bacteriocin structural genes (*ent*A, *ent*B, *ent*P, *ent*Q, *ent*AS-48, *ent* L50A/L50B and *bac*31) were studied by PCR in all bacteriocin producer (Bac+) enterococci using the primers and conditions previously described (10).

The genetic relationship among enterococcal strains was determined by pulsed field gel electrophoresis (PFGE) using *Sma*I enzyme as previously described (44). PFGE patterns were checked both visually and by numerical analysis after conversion and normalization. Band pattern similarity analysis was performed using GEL-Pro 3.1 software (Media Cybernetics, USA). PFGE patterns were clustered by the unweighted pair group method using arithmetic averages as determined with the MVSP 3.13I software program (Kovach Computing Services). A cutoff value of 85% identity was defined.

Multilocus sequence typing (MLST) was performed in 5 selected *E. faecium* isolates according to Homan *et al.* (19). The generated sequences were analyzed to determine the corresponding sequence type (ST) and clonal complex (CC) at the URL (http://mlst.ucc.ie/).

Sixty-three enterococcal isolates were recovered from the 31 samples of olive rhizospheres tested. Biochemical and molecular identification showed the presence of only two enterococcal species: *E. faecium*, which represented most of the isolates (61 isolates, 97%) and *E. durans* (2 isolates, 3%).

Our study showed a low percentage of resistance to erythromycin (3.2%). The presence of antibiotic resistance genes was studied by PCR in the two erythromycin-resistant *E. faecium* isolates, but they did not harbor the *erm*(B), *erm*(A), *erm*(C), *erm*(T) or *msr*(A) gene.

Tetracycline-resistance was detected in 7 isolates (11.2%) (6 *E. faecium* and 1 *E. durans*) and the genes *tet*(L) and/or *tet*(M) were demonstrated in all of them [*tet*(M) in 4 *E. faecium* and *E. durans* isolates and *tet*(M)+*tet*(L) in 3 *E. faecium* isolates].

Our isolates also showed a high frequency of resistance to ciprofloxacin (44.5%), but none exhibited a high level of resistance to gentamicin or streptomycin.

Three *E. faecium* isolates presented MICs for vancomycin between 8 and 16 μg mL^−1^, in the range of the intermediate resistance category according to CLSI 2010. They also appeared intermediately resistant to teicoplanin by the disk diffusion test. PCRs for *van*A, *van*B, *van*C1, *van*C2/3 and *van*D genes were negative in these three isolates. The amplicon obtained with degenerated *van*V1V2 primers was sequenced but corresponded with the specific ligase of the *E. faecium* species. In addition, no *van* genes were identified when primers *van*V3/*van*V4 and N*van*F/N*van*R were used in PCR reactions.

The *hyl* gene, encoding a glycosil-hydrolase, was identified in two tetracycline-resistant *E. faecium* isolates and one showed the intermediate phenotype of vancomycin resistance. The *esp* gene, encoding an enterococcal surface protein, was not observed among our enterococci.

All 61 *E. faecium* and 2 *E. durans* isolates identified were analyzed by PFGE using the *Sma*I restriction enzyme, detecting the high diversity of PFGE patterns among them. Indeed, 55 *E. faecium* and 2 *E. durans* exhibited 44 different pulsotypes, when considering that 85% is the homology level of group differentiation. The remaining 6 *E. faecium* isolates were not typable by PFGE despite repetition of the experiment (Fig. 1).

Furthermore, MLST was performed on five selected strains (3 vancomycin intermediate-resistant strains and two erythromycin strains). The obtained results revealed that two vancomycin intermediate-resistant *E. faecium* strains were classified by MLST analysis into sequence types ST21 and ST606. Interestingly, new sequence types (STs) were identified for three *E. faecium* isolates (2 erythromycin-resistant isolates and one vancomycin intermediate-resistant isolate, which also harbored the gene *hyl*). The new detected sequences were registered on the MLST website (http://efaecium.mlst.net/) as ST613, ST614 and ST615 (Table 1).

Forty-one percent of isolates (*n*=26) showed antimicrobial activity against at least one of the tested indicator bacteria. All belonged to the *E. faecium* species. Twenty of the 26 bacteriocin-positive (Bac+) *E. faecium* strains revealed the presence of the genes *entA* and/or *entB* (*entA*: 2 strains; *entB*: 14 strains; *entA*+*B*: 4 strains), encoding enterocins A and B, respectively.

In the current study, we reported the isolation and characterization of enterococci from rhizosphere samples. Although their frequent association with fecal and food samples, different studies have reported the occurrence of enterococci in soils (2, 8, 15, 32, 34). In accordance with our results, some of these studies also showed the predominance of the species *E. faecium* among the material which is in direct contact with the rhizosphere but in the presence of other species (2, 32). The predominance of this species is probably due to its broad spectrum of natural and acquired resistance and its ability to survive heat treatments and adverse environmental conditions (17, 23). Besides *E. faecium*, there was a small proportion of *E. durans*. Indeed, according to the classification of Kholer *et al.* (24) and Cools *et al.* (9), this species was revealed to belong to the *E. faecium* group, as they exhibited the same phenotypic properties of resistance to hostile environmental conditions (salinity and desiccation, among others). Fecal bacteria are known to have a limited lifespan in the environment, which could partially explain the absence of other enterococcal species characterized by their fragility once released into the soil (20). *E. faecium* seems, however, to be more adapted to such an environment and its presence could be supported by the availability of root exudates in this specific ecosystem closet to the plant roots (22). Nevertheless, many ecological questions related to its role in soils and plants remain unclear.

A low frequency of resistance to antibiotics (erythromycin and tetracycline) was detected among rhizospheric enterococci compared to clinical and animal isolates (2, 38), with a frequency higher than 50% for erythromycin and tetracycline. These results are similar to those reported by Muller *et al.* (32) for plant-associated enterococci and could be explained by the nature of the soil (non-agricultural soils) from which the samples were obtained. In fact, a major route of the dissemination of antibiotics to the environment is the application of slurry from treated farm animals to soils. Manure might contain fecal bacteria carrying resistance genes, possibly on mobile genetic elements that could be transferred to other soil bacteria (3, 9). Others mechanisms of resistance to macrolides could be present in erythromycin-resistant isolates, contrary to other studies (20, 37) indicating that *erm*(B) is the most frequent resistance gene found among erythromycin-resistant enterococci from the environment, food, animals and humans. Some authors have postulated that antibiotic resistance genes could be intrinsic in some soil bacteria with other functions, as is the case of metabolic or intercellular communication functions, and deviation from the primary role of these genes could be a response to the selective pressure of antibiotics (29).

We noted 7 tetracycline-resistant isolates. The overuse of tetracycline in clinical therapy and animal husbandry allows the accumulation of antibiotics in soils receiving animal waste as fertilizer and consequently the emergence of resistant bacteria by horizontal transfer of mobile genetic elements (3).

Interestingly, we detected four strains intermediately resistant to vancomycin, for which the mechanisms of resistance are still unknown and need to be studied in the future. In fact, Kuhn *et al.* (25) did not detect vancomycin-resistant enterococci in soil from different European regions, but only in sewage water; however, Guardabassi *et al.* (18) indicated that soil is a rich reservoir of genes conferring glycopeptide resistance in clinical bacteria.

The overuse of antibiotics related to the family of fluoroquinolones in veterinary therapy probably contributed to the selection of resistant bacteria to ciprofloxacin. The mechanism of resistance is mainly associated with mutations in *gyr*A and *par*C genes causing the low affinity of quinolones for their targets (6). The high frequency of resistance to trimethoprim-sulfamethoxazole (100%) is explained by the fact that this antibiotic has low intrinsic activity against enterococci.

The virulence genes *hyl* and *esp* were not detected among our enterococcal isolates, except for two strains that harbored the *hyl* gene. According to Laverde Gomez *et al.* (27), this gene is located on a megaplasmid, which is widely distributed among clinically associated *E. faecium* and seems to be restricted to this species.

The high rate of antimicrobial activity among the isolates is in agreement with those already reported by Del Campo *et al.* (11) and De Vuyst *et al.* (10). The majority of our isolates showed the ability to produce bacteriocins, which was partially explained by the presence of the genes *entA* and *entB*. However, for Bac+ strains and those negative for all bacteriocin tested genes, antimicrobial activity could be related to new enterococcal bacteriocins or other non-tested known genes. Enterococcal strains that produce bacteriocins seem to have an ecological advantage when compared with other non-producing bacteria which inhabit the same ecosystem (38), or which compete for colonization and invasion in a particular ecological niche. In addition, enterococci are good acidifiers in the presence of available sugars, and the inhibitory effect owing to acid production could be considered an advantage in addition to bacteriocin production, and is equally responsible for survival and colonization.

We note that enterococci in rhizospheres are characterized by high genomic diversity, detected by PFGE patterns. In addition, the MLST method has demonstrated new genetic lineages of *E. faecium* among rhizosphere isolates. Meanwhile, sequence type ST21 was also reported by Homan *et al.* (19) in two *van*A-containing *E. faecium* strains isolated from a cat and a hospitalized human. Environmental enterococci could therefore be considered as important source of original emergent types exhibiting new mechanisms of antibiotic resistance and as good candidates and suppliers of antimicrobial compounds.

## Figures and Tables

**Fig. 1:**
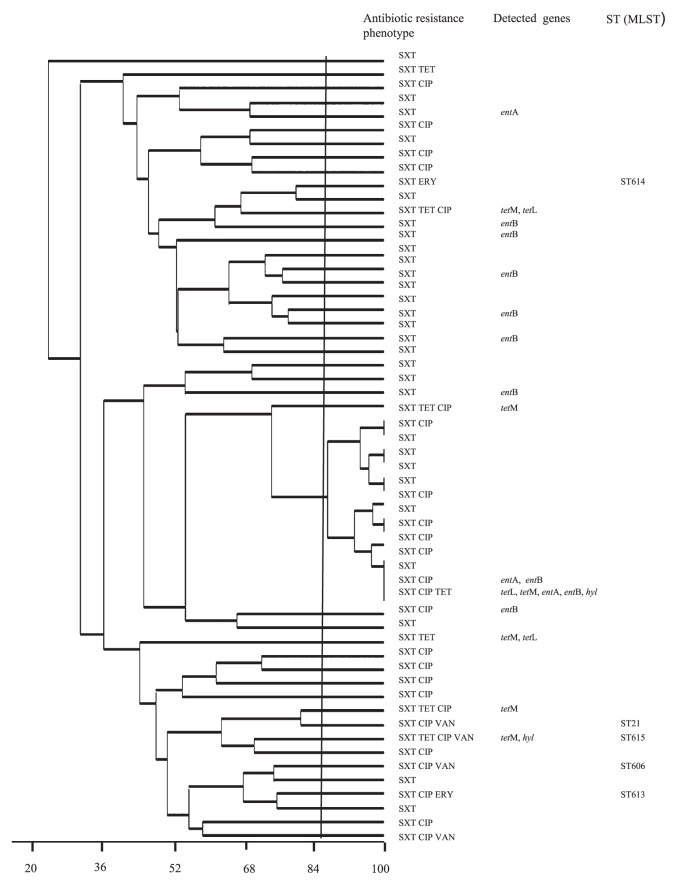
Dendrogram obtained by UPGMA showing the different pulsotypes, the new mutilocus sequence types (MLST), resistance phenotypes and detected genes related to antibiotic resistance, bacteriocin and virulence among enterococci isolated from rhizospheres. ERY, erythromycin; CIP, ciprofloxacin; TET, tetracycline, SXT, trimethoprim-sulfamethoxazole.

**Table 1:** Characteristics of the five *Enterococcus faecium* isolates selected for mutilocus-sequence-typing analysis (MLST)

Strain	Phenotype of resistance	*atp*A	*adk*	*ddl*	*gyd*	*gdh*	*pur*K	*pst*S	Sequence Type (ST)	Genes detected
*E. faecium*	Ery/Sxt/Cip	6	6	6	3	14	4	46	ST613 (new)	
*E. faecium*	Sxt/Cip/Van*	2	1	7	1	1	6		ST606	*ent*A, *ent*B
*E. faecium*	Sxt/Cip/Van*	9	1	3	1	1	2	1	ST21	
*E. faecium*	Ery/Sxt	10	6	13	17	9	17	19	ST614 (new)	
*E. faecium*	Sxt/Cip/Tet/Van*	5	1	40	2	12	3	1	ST615 (new)	*tet*M, *hyl*

Abbreviations and symbols: Ery, erytromycin; Cip, ciprofloxacin; Van*, vancomycin, intermediate resistance; Tet, tetracyclin, SXT, Trimethoprim-Sulfamethoxazol; ST, sequence type; *ent* A, gene coding for enterocin A; *ent* B, gene related to enterocin B; *hyl*, gene coding for glycosil-hydrolase; *atp*A, *adk*, *ddl*, *gyd*, *gdh*, *pur*K and *pst*S, housekeeping genes for MLST of *E. faecium*.
